# From Transgenesis to Genome Editing in Crop Improvement: Applications, Marketing, and Legal Issues

**DOI:** 10.3390/ijms24087122

**Published:** 2023-04-12

**Authors:** Daniela Marone, Anna Maria Mastrangelo, Grazia Maria Borrelli

**Affiliations:** Council for Agricultural Research and Economics, Research Centre for Cereal and Industrial Crops, 71122 Foggia, Italy; daniela.marone@crea.gov.it

**Keywords:** breeding, transgenesis, genome editing, biotech crops, biotech regulation

## Abstract

The biotechnological approaches of transgenesis and the more recent eco-friendly new breeding techniques (NBTs), in particular, genome editing, offer useful strategies for genetic improvement of crops, and therefore, recently, they have been receiving increasingly more attention. The number of traits improved through transgenesis and genome editing technologies is growing, ranging from resistance to herbicides and insects to traits capable of coping with human population growth and climate change, such as nutritional quality or resistance to climatic stress and diseases. Research on both technologies has reached an advanced stage of development and, for many biotech crops, phenotypic evaluations in the open field are already underway. In addition, many approvals regarding main crops have been granted. Over time, there has been an increase in the areas cultivated with crops that have been improved through both approaches, but their use in various countries has been limited by legislative restrictions according to the different regulations applied which affect their cultivation, marketing, and use in human and animal nutrition. In the absence of specific legislation, there is an on-going public debate with favorable and unfavorable positions. This review offers an updated and in-depth discussion on these issues.

## 1. Introduction

To cope with human population growth and climate change, there is a need for modern breeding programs to identify new sources of genetic diversity to improve adaptation to abiotic or biotic stresses and to yield performance and quality traits. Since the end of the last century, decisive knowledge of plant genomes has helped to reach these goals.

Conventional breeding is still heavily based on the genetic diversity naturally present in crop species and it receives strong support from the use of molecular markers closely linked to useful alleles for desired traits. Through marker-assisted selection (MAS) it is possible to select parental lines suitable for crosses aimed at introducing useful genes into a new progeny to improve the effectiveness or efficiency of selection for the traits of interest [1]. Recent advances in marker technology, including high-throughput genotyping, have increased the utility of MAS or pyramiding for breeding, even though selection from conventional crosses is very time-consuming, requiring at least 5–7 cycles of backcrossing. Novel genetic variation can also be created using mutagenic chemical/physical approaches, such as the use of ethyl methanesulfonate or high energy radiation to alter the genome of a given crop [2]. Mutation breeding has some drawbacks. In fact, mutations are introduced randomly and are uncontrolled, and a large mutant population must be generated and screened to identify interesting genetic variation [3]. Moreover, several genes can be mutated at the same time, creating undesired genetic changes that require multiple rounds of backcrossing to isolate the desired mutation in the new genetic background. For all these reasons, this process is slow and very long, requiring 8–10 years.

In the last century, transgenic breeding has been developed to create genetic variations in crops, which involves the addition of foreign genes to plant genomes [4]. Transgenic research has improved and gene transfer from wild relatives into crops has accelerated, overcoming the reproductive barriers among different plant species. It is an accurate and precise tool for improving crop tolerance to a wide range of stresses without problems related to linkage drag. The success of transgenic breeding is mainly linked to herbicide tolerance and insect resistance, for which transgenic lines have been adopted worldwide; other transgenic events are at a pre-commercial phase and many more are still at the laboratory level and most likely a few of them will be successfully validated in field trials. Although advances in genomics and bioinformatics in many crops are fueling the discovery and cloning of genes for improved agronomic traits of interest, and thus, providing exciting opportunities for genetic engineering of desired characteristics in plants, many ethical and legislative problems related to environmental safety and health severely limit the development, commercialization, and use of genetically modified (GM) crops in many countries in the world. Over the past 20 years, sequence-based knowledge coupled with advanced biotechnologies have supported the development and widespread application of new plant breeding techniques (NBT) to obtain the best results regarding the modification and transfer of useful alleles in elite varieties and to accelerate crop improvement together with great public acceptance. Among these, a useful opportunity has been provided by the development of gene-editing (GE) technologies that allow genetic variation to be introduced with a high degree of precision and specificity, only in the gene of interest [5,6,7]. GE technologies also provide the possibility of multiplexing, which simultaneously causes targeted mutations in multiple genes [8]. Since GE is more efficient and potentially safer, it can speed up the process of generating varieties with traits of interest, overcoming many of the objections related to transgenesis [9]. The extent to which GE technologies can develop will largely depend on how GE-edited crops are internationally regulated and whether they are subject to the same costly law that applies to GM organisms (GMO). 

**Figure 1:**
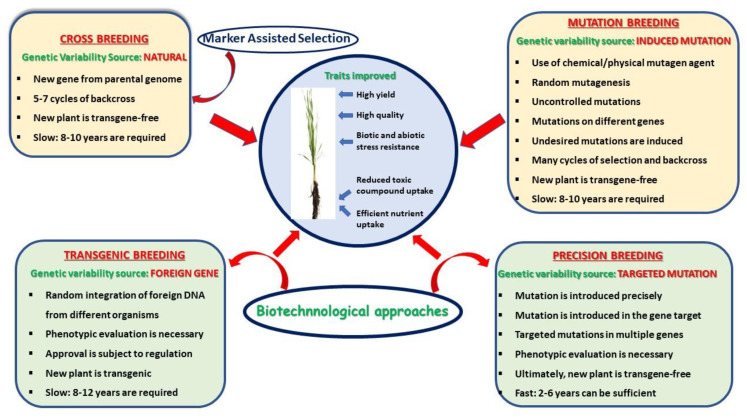
Traditional and innovative plant breeding techniques using different sources of genetic variability to introduce new traits into an elite crop variety.

An important aspect in choosing a breeding approach is the work and time needed to develop the necessary knowledge prior to the stage of selection (Figure 1).

Traditional breeding that is based on randomly induced mutations requires less effort and use of technologies in the initial phase of the program, but a consistent and long effort is needed to select the genotypes that carry the traits of interest in a large progeny, since the selection is mainly based on phenotype. MAS requires a longer preliminary study to identify the genetic association between the phenotypic traits and molecular markers, but then it can significantly accelerate the selection phase. Biotechnological approaches based on transgenesis require an even longer initial development of the knowledge of the genetic basis of the various traits, with the identification of the causal genes/alleles controlling the trait. Indeed, for transgenesis, the gene has to be identified, also in sexually incompatible species, cloned, and inserted into the constructs to be used in the various methods of genetic transformation. In GE, the gene sequence must be known to allow the induction of the desired targeted mutations, and ad hoc constructs are prepared for transformation. In both cases, the genetic transformation may be the limiting step, in particular for some species/genotypes. Therefore, it is necessary to optimize the entire process which allows, through the various stages of in vitro culture and selection, to obtain new regenerated fertile plants with the new traits of interest. 

The diffusion of biotech crops, meaning both GM crops and those obtained through GE, is on the rise globally due to the enormous benefits to the agronomic traits (increasing the yield and maintaining biodiversity), the environment (mitigating the challenges associated with climate change, reducing chemical input, and CO_2_ emissions), the health of humans and animals, and the socioeconomic conditions of farmers and consumers [10]. Biotech crops are rapidly occupying a large part of global trade, despite different regulations around the world, ranging from moratoria and bans to regulations that treat conventional and biotech products under the same regulatory framework.

In this review, we present an updated overview of the research on biotech crops, namely transgenic and GE-edited crops. In particular, the more advanced studies where phenotypic evaluations in the open field have been carried out are considered in order to discuss the most promising genetic modifications based on their real impact on crop cultivation, and their diffusion in the world. Regulations applied by various countries which affect their cultivation, marketing, and use in human and animal nutrition are discussed in light of future developments and applications.

## 2. Improvement of Agronomic Traits through Transgenic Approaches

### 2.1. Resistance to Herbicides

To date, many studies have been conducted to improve plant tolerance to abiotic and biotic stresses and quality-related traits through genetic transformation and have resulted in several important achievements, although only a few studies have reported examples of field trial evaluations (Table S1). Recently, Ricroch et al. [11] reviewed biotechnological plants, including transgenic cotton, cowpea, maize, soybean, potato, and rice, that had been marketed and approved for cultivation (in at least one country). The case of the glyphosate-resistant wheat developed by Monsanto and authorized for field tests in 16 states in the United States (USA) from 1998 to 2005 is well known, even if it is not currently marketed, probably because of potential market loss due to consumer reluctance. In 1996, glyphosate-resistant soybean became the first herbicide-resistant crop to be marketed in the USA [12], and it is now currently grown in many other countries including Argentina and Brazil [13], which has made it a leading biotech crop. This soybean variety allows for growers to spray herbicides to kill any weeds in the field while not killing the soybean crop (reviewed by Xu et al. [14]). Another successful example of resistance to herbicides has been reported in pineapple [15]. Transgenic plants transformed with the *bar* gene for bialaphos herbicide resistance were developed and evaluated for tolerance to the herbicide ”Basta”. Seven months after transfer to the field, transformed pineapple plants were found tolerant to 1600 mL/rai of the herbicide, which was twice the dose recommended for field application of the herbicide itself. As the application of herbicides is considered to be a necessary part of modern agriculture to combat undesired weeds causing crop losses of 20–60%, this type of approach could accelerate the growing of herbicide-tolerant crops with benefits in terms of yield and costs, even if the adoption of herbicides does not meet the principles of agricultural sustainability. 

### 2.2. Tolerance to Abiotic Stresses

Overexpression of ROS scavenging enzymes via genetic transformation has been used as a potential strategy to overcome heat stress in tomato. Wang et al. [16] developed transgenic tomato plants which overexpressed the *cytosolic ascorbate peroxidase (cAPX)* gene with enhanced tolerance to heat (40 °C). Indeed, detached fruits from field-grown transgenic tomato plants showed enhanced resistance with exposure to direct sunlight as compared to fruits from wild-type (non-transgenic) plants. This demonstrates that the transgenic technology of such genes may be able to increase the tolerance of plants to oxidative stress and to improve their performance under field stress conditions. An enhanced resistance to high temperatures in the field has also been achieved in Chinese cabbage (*B. rapa* ssp. *pekinensis*) through the overexpression of the Arabidopsis gene *HTT2* (*HEAT-INDUCED TAS1 TARGET2*) [17]. Transgenic plants with *HTT2* appeared greener in the field and formed leafy heads earlier than wild-type plants. Exogenous *HTT2* increased the survival rates of heat-shocked heading Chinese cabbage by promoting thermotolerance through decreasing electrical conductivity and extending hypocotyl length. Moreover, stable dehydration-inducible *GmMYB14*-overexpressing transgenic soybean plants demonstrated semi-dwarfism and a compact plant architecture associated with drought tolerance under field conditions together with improved yield [18]. 

Finally, transgenic wheat (*Triticum aestivum* L.) expressing a mutated version of the HaHB4 transcription factor from *Helianthus annuus* (*Hahb-4*) showed improved yield and increased drought tolerance when evaluated in multiple field trials with respect to the wild type [19]. The HB4 wheat developed by Bioceres, S.A. has been approved for food and feed direct use or processing in many countries, such as Brazil, Australia, Columbia, New Zealand, Nigeria, USA, and in Argentina also for cultivation [20]. 

### 2.3. Resistance to Biotic Stresses

Various biotic stresses (resistance to some insects, nematodes, bacterial or fungal diseases, and viral diseases) have been improved in transgenic plants obtained either by classical gene transfer (via *Agrobacterium* or biolistic approaches) or RNAi gene silencing. A larger number of studies that have reported results on improved crops for biotic stresses are available in the literature compared to those aimed at the development of crops improved for abiotic stresses [21,22]. In fruit crops, a coat protein-mediated approach to engineer virus resistance has been applied to introduce resistance against various viral diseases. For example, transgenic papaya plants with the mutated *replicase* (*RP*) gene from PRSV (papaya ring spot virus) has shown high resistance or immunity against PRSV in the field [23]. Dutt et al. [24] overexpressed the *Arabidopsis thaliana NPR1* gene, playing a key role in regulating salicylic acid (SA)-mediated SAR in plants, under the constitutive promoter *CaMV35S* and also under a phloem-specific Arabidopsis *SUC2* (*AtSUC2*) promoter in sweet orange cultivars “Hamlin” and “Valencia”. The transgenic plants obtained exhibited reduced disease severity, and a few lines remained disease-free even after three years of planting in a high-disease pressure field site. In regard to cereal crops, in rice, a dominant gene of the wild species *Oryza longistaminata* (*Xa21*) conferring resistance to all known races of the bacterial blight pathogen *Xanthomonas oryzae* pv. *oryzae* was transferred by transformation to susceptible lines and one of the transgenic lines revealed excellent agronomic traits in field tests [25]. Therefore, this type of approach seems to be very promising for developing varieties with resistance to multiple races/species that are able to have good performances in the field; however, many countries have severe rules against biotech crop cultivation in the open field. Moreover, a lot of work has been done to make transgenic rice by introducing the gene *cry1Ab* of the bacterium *Bacillus thuringiensis (Bt*), as the best method to obtain plant resistance against pest invasions and to reduce the environmental damage caused by chemical pesticides. These lines were tested in a field experiment in three isolated regions under the biosafety standard protocol in northern Iran, in 2016 [26]. The findings demonstrated that, in all three regions, transgenic lines with an active *cry1Ab* gene derived from the ”Khazar” cultivar were similar to their parental lines in terms of growth phenology, as well as agronomic and quality-related traits, but showed lower yield loss and better yield-related traits compared to their non-transgenic parental lines in the presence of artificial inoculation of the disease. 

Many studies based on genetic transformation have been reported on sugarcane. Transgenic sugarcane plants with improved resistance to either biotic or abiotic stresses, were obtained through both *A. tumefaciens* and biolistic methods [27], even if there are very few officially approved varieties for commercialization, with insect resistance, and recently released in Brazil. Some studies have observed reduced phenotype expression for agronomic traits of interest, for example, the transgenic sugarcane line that showed both insect resistance and glyphosate resistance in field conditions presented poor agronomic and industrial traits compared to non-transformed control plants [28]. Moreover, field studies are available on disease-resistant transgenic sugarcane, such as the two cultivars transformed for resistance to the sugarcane mosaic virus (SCMV) strain E, for which a large variation in both yield characteristics and disease resistance was found in the field [29]. In a three-year study, transgenic lines transformed for sugarcane yellow leaf virus (SCYLV) resistance were evaluated for agronomic traits of interest and virus resistance, and the transgenic lines showed reduced performance compared to the parental genotype [30]. In contrast, a good result was reported for transgenic sugarcane lines expressing the coated protein gene *CP* of SCMV for which a greater yield and a lower SCMV disease incidence was observed in four experimental locations in China across two successive growing seasons [31]. 

Interestingly, a lot of work has been carried out to improve resistance to *Phytophthora infestans*, a pathogen that causes a very difficult disease to control, with severe damage to the foliage of tomatoes and tubers of potatoes. To date, single *Rpi* genes conferring resistance to this fungus have been introduced into several potato cultivars. Transformed cv. ”Désirée” plants with the *Rpi-vnt1.1* gene remained fully resistant to *P. infestans* or had reduced disease severity compared to susceptible controls when tested in field experiments [32]. Moreover, a significantly higher level of resistance was achieved by introducing three *Rpi* genes (*RB*, *Rpi-blb2,* and *Rpi-vnt1.1*) [33]. The same *Rpi* genes were also stacked in two popular Kenyan potato cultivars ”Tigoni” and ”Shangi” [34].

### 2.4. Improvement of Quality-Related Traits

The well-known example of transgenic soybean characterized by improved oil quality (oleic acid content), which is beneficial for human health, was obtained by RNAi-mediated knockdown of *Glycine max fatty acid desaturase2-1B* (*GmFAD2-1B)*, coding for a key enzyme responsible for converting oleic acid (18:1) precursors to linoleic acid (18:2) in the lipid biosynthetic pathway [35]. Unfortunately, the total protein and oil contents and agronomic traits of the transgenic lines evaluated in field trials did not show a significant difference compared with the wild type plants. Another example is represented by the sweet potato orange (IbOr) protein involved in the accumulation of carotenoids introduced into purple-fleshed sweet potato plants to produce both anthocyanins and carotenoids in their storage roots [36]. The carotenoid contents and transcription levels of carotenoid biosynthetic pathway genes in the transgenic plants were enhanced. The plants were evaluated under field conditions and, in this case, no difference was also observed by comparing the yields of storage roots and aerial parts of transgenic versus wild-type (WT) plants. 

In addition, a study of the impact of endogenous lipids on baking through transgenic modification has been reported in wheat. A general consensus exists in the literature that removing or modifying triacylglycerides (TAG) to more polar forms is more desirable during processing, while Frauenlob et al. [37] showed that the addition of various lipases to flour, with activity against both polar lipids and TAG, could increase loaf volume. Transgenic wheat with increased endosperm lipid has been produced with the introduction of three genes (endosperm-specific promoters driving maize *Wrinkled1a*, Arabidopsis *diacylglycerol acyl transferase 1* (*DGAT1*), and sesame *oleosin* to determine the impacts on grain composition and baking quality [38]. Indeed, these lines consistently produced grain with a substantive increase of triacylglycerides (TAG) levels, up to eight-fold, in the endosperm over five generations, including two seasons in the field, with no change in polar lipid content and few changes in the composition of whole meal flours obtained by field-grown grain. Moreover, even if seedling growth or grain yield were not significantly diminished in the field, average individual seed weight and diameter were slightly reduced, and small-scale baking of field-grown flours showed a small decrease in bread loaf volume but no significant change in biscuit diameter or height. 

A positive effect on the agronomic traits of interest, together with the effectiveness of the new trait introduced, has been highlighted in field trials carried out to assess transgenic lines of canola overexpressing the *acyl-CoA-dependent diacylglycerol acyltransferase* (*DGAT1*) gene under the control of the seed-specific promoter *napin*. In field trials, transgenic lines exhibited increased seed oil content and performed quite well for typical agronomic traits, as well as protein and glucosinolate content [39].

Finally, an interesting study on the concentration of zinc in cereals, a very important trait for human nutrition, reported that transgenic barley lines expressed *cytokinin oxidases/dehydrogenase (CKX*), a cytokinin-degrading gene, in their roots; thus, increasing the degradation of this hormone and making favorable root elongation and branching, characterized by an increased concentration of Zn in grains up to 30%, also under field growth, opening the perspective of developing a sustainable strategy for biofortification of cereal grains, especially if confirmed by multilocation trials [40].

With regard to biotechnology applied to horticulture, the first field trials of transgenic plants were conducted in France and USA, in 1986, with improved shelf-life trait for the transgenic Flavr Savr tomato [41]. This is an example of the production of transgenic crops with commercial value. In fact, the ”Flavr Savr” tomato was obtained by silencing the endogenous *polygalacturonase* (*PG*) expression using RNAi technology, and it was approved for commercialization in 1993 by the U.S. Food and Drug Administration (FDA) [42]. 

## 3. Improvement of Agronomic Traits through Intragenesis/Cisgenesis

To overcome objections to transgenesis, two eco-friendly transformation approaches, intragenesis and cisgenesis, have been developed for transferring genes with their regulatory sequences, from the same species or from crossable, sexually compatible species [43,44,45,46,47]. These approaches can replace inherent alleles with superior ones or can introduce new genes into the cultivated gene pool. The goal is to transfer genes of interest either from related species or the overexpression of those already present within the crop itself, avoiding linkage drag that occurs when gene transfer is obtained by crossing. Thus, the gene pool exploited by these approaches is identical to the gene pool available for traditional breeding. At the basis of these approaches, there is the need to isolate the complete functional genes, together with their associated promoter/terminator (regulatory sequences). This is facilitated by the advancement of sequencing technologies and the availability of wide genome information. Unfortunately, they are limited to a few crop species, such as wheat [48,49], barley [50], horticultural, fruits, and ornamental crops [22,45,51]. Moreover, very few examples have been reported that have been tested in field trials (Table S1). Cisgenic “Désirée” potato plants with two *Rpi* genes (*Rpi-blb3/RPI-sto1*, *Rpi-vnt1:/Rip-ch1,* or *Rpi-vnt1/Rip-sto1*) and three *Rpi* genes (*Rpi-blb3/Rip-vnt1/Rip-sto1*) remained fully resistant to *P. infestans* or had reduced disease severity compared to susceptible controls under natural infection, during two-year field tests [52]. Cisgenic cv. “Désirée” with three *Rpi* genes (*Rpi-blb3*, *Rpi-vnt1.1,* and *Rpi-sto1*) was also obtained by Haesaert et al. [53], showing complete resistance to late blight, during two-year field trials in Belgium and the Netherlands. Krause et al. [54] analyzed the impact of a cisgenic modification of the potato variety Desirée to enhance resistance against the late blight-causing fungus *Phytophthora infestans* (Oomycetes) on the abundance and diversity of rhizosphere microbial communities in two field locations. By comparing the cisgenic version of Desirée in the presence and absence of fungicides to its non-engineered late blight-sensitive counterpart, the authors demonstrated environmental variation but also similar patterns of soil microbial diversity in potato rhizospheres and indicated that the cisgenic modification had no tangible impact on soil microbial communities. In the same crop, silencing of the *StAst1* gene involved in asparagine biosynthesis to limit acrylamide in French fries generated intragenic potatoes with 70% reduction in acrylamide levels after processing, and field trials with these potatoes showed a normal tuber phenotype [55]. High-amylopectin potatoes [56], potatoes with resistance to late blight [52,53,54], apples with increased resistance to scab [57,58], and barley with improved phytase activity have been evaluated in field trials in the EU [50], while potatoes with improved processing qualities [55] have underwent field trials in USA [45]. 

Despite the greater value performance of the genotypes obtained by cis-/intragensis, further efforts are needed to overcome the limits of their wider application, such as the variability of gene expression or the silencing of endogenous genes depending on the random integration of the cis-/intragene or the presence of extraneous sequences [45]. 

## 4. Improvement of Agronomic Traits through Genome Editing (GE)

Although the first decade of GE research mainly focused on the setting up of the clustered regularly interspaced short palindromic repeats (CRISPR)/CRISPR-associated protein (CRISPR/Cas) editing system by making various technical improvements in its applications, recently, the number of scientific publications describing GE-edited plants for different traits of interest has been growing at a very fast speed [59]. An update on the general situation of GE crops was reported by Hamdan et al. [59] and Menz et al. [60]. Menz et al. [60] analyzed the results of 231 studies that had been carried out by public institution and private companies, involving 25 different countries, mainly China, the USA, Japan, Germany, France, and the UK. Until now, two model plants (Arabidopsis and tobacco) and 41 crop plants and ornamentals have been edited, mainly rice, tomato, and maize, followed by wheat, potato, soybean, and ornamentals [59,60]. To a lesser extent, studies on peanut, kiwi, lettuce, lemon, poppy, salvia, cacao, banana, manioc, and sugarcane have also been performed [60]. Among the different GE applications with identified targeted trait improvements, most concern relevant agronomic traits (growth performance, storage performance, yield increase, evaluated as grain weight, size, and number), followed by food/feed quality and nutritional traits (e.g., oil composition, better digestibility, increased vitamin content or reduced starch, phytic acid, and allergen content), traits for biotic stress tolerance (to virus, fungi, and bacteria), herbicide tolerance and, to a minor extent, abiotic stress resistance (drought, salt, and heat/cold tolerance), ornamentals (growth performance), and industrial applications (product quality and growth performance). In addition, multiple traits (growth performance/quality/yield increase/herbicide tolerance) have been simultaneously edited [59,60,61]. GE-edited lines have been phenotypically evaluated and appreciable effects of induced gene mutations have been observed in many cases. Nevertheless, only in a few cases have the obtained lines been evaluated in field trials, to provide strong indications on the effective impact of the GE-induced mutation on the agronomic traits of lines (Table S1). There are several reasons to explain the limited phenotypic evaluation of GE-edited lines: a large amount of seed is needed to arrange a trial with plots big enough for the correct evaluation of agronomic traits such as grain yield; field trials are more complex to manage for research groups compared to experiments in controlled conditions; and, more importantly, in many countries, such as those of the European Union (EU), GE-edited plants are considered by law to be GM organisms, and their growth in open field is forbidden. Mainly for this reason, most of the reports in which GE-edited plants have been evaluated in field trials have originated from China and USA [62]. The scientific community is now asking governments of countries in which GE-edited plants are considered by law to be GM organisms, to consider a different regulation since, following some self-crossing and selection cycles, it is possible to obtain plants completely devoid of foreign DNA, and carrying only the desired mutations in the target gene(s). Some of the studies available in the scientific literature have shown that GE techniques are able, therefore, to produce plants with phenotypes absolutely similar to those obtained through traditional (natural or chemically/physically induced) mutations in the same genes. This is very important in crops, such as grapevine and olive, where varietal identity is covered by license.

### 4.1. Improvement of Yield and Yield-Related Traits

Li et al. [63] used a CRISPR/Cas9 system to mutate four genes in the rice cultivar ”Zhonghua 11”, previously reported to function as regulators of grain number (*Gn1a*—Os01g0197700), panicle architecture (*DEP1*—Os09g0441900), grain size (*GS3*—Os03g0407400), and plant architecture (*IPA1*—Os08g0509600). Mutated plants showed interesting phenotypes, comprising enhanced grain number (*gn1a*), dense erect panicles and semi-dwarf phenotype (*dep1*), and larger grain size and grain with long awn (*gs3*). The *ipa1* mutants showed two contrasting phenotypes, having either fewer tillers or more tillers, depending on the changes induced in the *OsmiR156* target region. All of these phenotypes were similar to those described in some previous reports for mutants obtained with different methods. Moreover, modifying multiple regulators of important traits in a single cultivar by using CRISPR/Cas9 can facilitate the dissection of complex gene regulatory networks in the same genomic background and the stacking of important traits in cultivated varieties. Indeed, single mutations can be combined through crosses into the same genotype. 

Another way to produce mutants in different genes is to use GE working on multiple genes at the same time to obtain a stronger effect on the desired phenotype. A vector targeting eight genes simultaneously using a CRISPR/Cas9 multiplex genome editing system was recently constructed to improve grain yield in rice [64]. Homozygous sextuple, septuple, and octuple mutants were identified, and some of them, tested in field conditions, showed improved yield-related traits such as grain length and width, and thousand kernel weight. Similarly, Zhou et al. [65] simultaneously targeted, in three elite rice varieties, three yield-related genes, *OsGS3*, *OsGW2* and *OsGn1a*, previously described to negatively regulate grain size, width and weight, and number, respectively. Seven combinations of single, double, and triple mutants for the target genes were developed, which showed an effect on traits such as grain length, width, number, and 1000-grain weight. Overall, the additive contributions resulted in increase of up to 68% yield per panicle in triple mutants in field conditions. These abovementioned results show that GE is a very promising tool for modifying multiple genes at the same time and for obtaining a strong phenotypic effect in field conditions.

Very interesting results have also been obtained related to improved yield. A CRISPR/Cas9 genome editing system was used to edit *Semi-Dwarf1* (*SD1*) to confer shorter plant height and better resistance to lodging in the landraces ”Kasalath” and ”TeTePu”, chosen for their tolerance to low phosphorous and broad-spectrum resistance to several diseases and insects [66]. Field trials in two locations demonstrated that the yields of the mutant lines were better than those of the wild-type plant under modern cultivation, while no differences were observed for other agronomic traits. In this case, grain yield was improved because of the reduction in plant height. In another study, a higher grain yield was obtained in common wheat by improving nitrogen-use efficiency. This goal was reached by targeting *TaARE1*, a homolog of the rice gene *ARE1* [67], involved in NUE, and, under nitrogen-limiting conditions, the rice *abnormal cytokinin response1 repressor1* (*are1*) mutant exhibited increased NUE, delayed senescence, and consequently, increased grain yield. All transgene-free mutant lines showed enhanced tolerance to N starvation in hydroponic culture and showed delayed senescence and increased grain yield in field conditions in three locations under conventional management. In addition to nitrogen starvation, another stress which strongly limits grain yield in the field is drought. Shi et al. [68] targeted the promoter of the maize gene *ARGOS8*, a negative regulator of ethylene responses, and obtained plants overexpressing the gene. A field study in six different locations showed that, compared to the wild-type plant, the *ARGOS8* variants increased grain yield by approximately 0.34 t ha^−1^ under flowering stress conditions and had no yield loss under well-watered conditions. 

### 4.2. Tolerance to Abiotic Stresses

When considering the studies published on GE-modified plants, field trials are important to demonstrate that the agronomic traits of the plants edited for a particular trait and evaluated in controlled conditions are similar to those of the wild-type plants. In this way, the absence of negative impacts of GE on agronomic traits can be shown. Zhang et al. [69] developed nine rice mutant plants edited for the *OsRR22* gene, by coding for a B-type response regulator transcription factor that is involved in both cytokinin signal transduction and metabolism, whose loss of function significantly increases salt tolerance [70]. Two mutant plants without transferred DNA (T-DNA) were phenotypically tested for salt stress tolerance at the seedling stage in controlled conditions, and showed an improved phenotype compared to the wild-type plants, while no significant differences were observed in agronomic traits when the plants were tested in the field in the absence of stress [69]. 

### 4.3. Improving Resistance to Plant Pathogens

Some examples are available of successful events of genome-edited plants that have shown resistance against plant pathogens. Zeng et al. [71] used a CRISPR/Cas9 system to disrupt the function of the sugar transporter *OsSWEET14* in the genome of rice cv. ”Zhonghua 11”. This gene is known to be a major susceptible gene of bacterial blight caused by *Xanthomonas oryzae* pv. *oryzae* (Xoo). Edited plants showed strong resistance to African Xoo strain AXO1947 and Asian Xoo strain PXO86. In the field, disruption of *OsSWEET14* function led to increased plant height without a reduction in yield. 

In other studies, field trials were used to directly test the effect of GE-driven mutations on the trait of interest. Zhou et al. [72] developed single and triple mutants by targeting three known broad-spectrum blast-resistant genes, *Bsr-d1*, *Pi21,* and *ethylene responsive factor 22* (*ERF922),* in rice. While all the single and triple mutants showed increased resistance to rice blast compared with wild-type plants, the *erf922* mutants displayed the strongest blast resistance similar with triple mutants probably due to the upregulation of genes associated with the SA- and JA-pathways. The *OsERF922* gene has been previously targeted by GE to improve resistance to rice blast [73]. In both studies, mutant plants were tested for disease reaction in both controlled and field conditions. Moreover, the field trials clarified that there were no trade-offs between resistances and main agricultural traits. These results are very interesting and open the way for a more durable and large evaluation of these mutant materials to assess the impact of such mutations in disease resistance over multiple geographical areas and multiple years. Indeed, a durable wide-spectrum resistance is expected for genes, such as *OsERF922*, which code not for nucleotide-binding domain leucine-rich repeat (NLR) receptors with a strict specificity in recognition of pathogen factors. 

### 4.4. Improvement of Quality-Related Traits

The number of environments in multi-location trials is important, since the impacts of GE-induced mutations are strong when observed in diverse environmental conditions. Recently, Gao et al. [74] developed waxy corn hybrids by using CRISPR/Cas9 editing of a *waxy* allele in 12 elite inbred maize lines. Field trials were carried out at 25 locations, and showed that CRISPR-waxy hybrids were agronomically superior to introgressed hybrids, producing on average 0.37 t ha^−1^ higher yield. 

Rice grain with excessive cadmium (Cd) is a major source of dietary Cd intake and a serious threat to health for people who consume rice as a staple food. Tang et al. [75] developed new indica rice lines with low Cd accumulation by knocking out the metal transporter gene *OsNramp5* using a CRISPR/Cas9 system. Edited plants accumulated less Cd compared to wild-type plants in hydroponic culture as well as in Cd-contaminated paddy field trials, without significantly affecting grain yield. A further investigation in which the same gene was edited in two japonica varieties revealed a mild reduction in grain yield, probably due to Mn deficiency which is carried by the same Cd transporter [76]. Finally, Songmei et al. [77] produced mutant lines for the genes *OsNramp5* and *OsLCT1*, a rice homolog of wheat *low affinity cation transporter 1*, localized to the plasma membrane, and found that mutants in *OsLCT1* could be used to produce rice grains that were safe for human consumption in lightly contaminated paddy soils, and mutants in *OsNramp5* could be used in soils contaminated by much higher levels of Cd. GE has been applied to study the minor uptake of toxic elements associated with the problem of radiocesium occurrence in food, particularly affecting the production of rice in Japan after the nuclear accident at Fukushima in 2011. Nieves-Cordones et al. [78] showed that inactivation of the Cs^+^- permeable *high-affinity K^+^ transporter* (*OsHAK1)* with the CRISPR/Cas system dramatically reduced Cs^+^ uptake by rice plants. Two experiments with contaminated soils from Fukushima indicated that inactivation of *OsHAK1* had no beneficial effect on rice ^137^Cs^+^ accumulation when its concentration in soil was very low, while it was a promising strategy to reduce ^137^Cs^+^ accumulation in plants grown in conditions favoring high radiocesium uptake by the plant.

The examples described so far have mainly been from rice and maize, due to laws preventing scientists in many countries from conducting field evaluations of GE-modified plants. Recently, Neequaye et al. [79] provided an example of GE-induced mutations of field-grown *Brassica oleracea*. They targeted the *MYB28* gene, which is involved in the regulation of aliphatic glucosinolate (A-GSL) biosynthesis and associated with sulfur metabolism. Based on the results of the first field trial with GE-modified plants in the United Kingdom (UK), approved and regulated by the UK Department for Environment, Food & Rural Affairs after the reclassification of gene-edited crops as genetically modified organisms by the European Court of Justice on 25 July 2018, the authors described the effect of knocking out *MYB28* on the downregulation of *A-GSL* biosynthesis genes and the reduction in accumulation of different methionine-derived glucosinolates, whereas accumulation of sulfate and indole glucosinolate in leaf and floret tissues remained unchanged. Furthermore, in 2021, Rothamsted Research in the UK received approval for field trials of genome-edited wheat modified to produce less asparagine, which is a compound that causes cancer when bread is toasted [80]. 

Moreover, field trials were carried out at Rothamsted Research to select, among the camelina lines obtained by GE of the *FAD2* gene for enabling the accumulation of oleic acid, those with the allelic combination able to ensure a high content of oleic acid without altering the agronomic performance traits [81]. 

The modification of lipid content in camelina and canola was also obtained by GE of the *biotin/lipoyl attachment domain containing* (*BADC*) gene, which is a negative regulator of the acetyl-CoA carboxylase (ACCase) that is the key enzyme to produce fatty acids for oil biosynthesis [82]. The results of field trials, carried out in 2021 by the Yield10 Bioscience Company in the USA, showed that both GE-edited camelina and canola produce increased in seed oil content comparable to that obtained in greenhouse studies [83]. Yield10 included these GE-edited crops in its 2022 field testing program to obtain additional oil content and seed yield data.

Finally, in the USA, Calyxt has developed a GE-edited high fiber wheat variety which has already been evaluated in field trials [84]. 

### 4.5. High-Throughput Approaches for Modifying Crops through GE

The results presented here point out that field evaluations of GE-modified plants, in particular in trials with many different locations, are very important to dissect the effect of such mutations on the agronomic performance traits of crops, indicating the most promising breeding strategies to follow. In the near future, a very interesting development would be to assess, in large field trials, a large number of different GE-modified lines, with different mutations in many different genes, to identify the most productive plants. In this regard, Meng et al. [85] realized a high-throughput CRISPR/Cas9 mutant library in rice that was useful for identifying gene functions of great potential for genetic improvement. More than 50,000 rice genes were initially identified and nearly 13,000 were selected for targeting, which were genes that are highly expressed in rice shoot. Two hundred T0 lines randomly sampled among 14,000 were phenotypically evaluated in the field, and interesting phenotypes which could be related to edited genes were identified. The mutant library was of high quality, with good coverage and uniform distribution, and it represents a very promising tool for functional genomics and breeding to also be realized in the near future in other crops.

## 5. Distribution Worldwide of Biotech Crops and Event Approval 

Biotech crops, mainly crops genetically modified for different traits, have been commercialized starting more than 20 years ago [86]. Global economic gains contributed by biotech crops from 1996 to 2018 amounted to USD 224.9 billion with economic benefits for almost 16–17 million farmers, most of whom are from developing countries [87]. A compound annual growth rate (CAGR) of 9.4% has been projected for 2022-2030, the USA still being the global leader in the development and commercialization of GM and GM crops [88] The global area of GM crops has increased from 1.7 million hectares in 1996 to 190.4 million hectares in 2019, distributed in 29 countries (24 developing and 5 industrial countries) [87]. The main GM crop-growing countries are the USA (with 95% of the entire cultivation area (mainly soybean, maize, and canola)), Brazil (94%), Argentina (~100%), Canada (90%), and India (94%). Nine countries in the Asia-Pacific region cover 10.2% of the total biotech crop area (India, China, Pakistan, Australia, Philippines, Myanmar, Vietnam, Bangladesh, and Indonesia), with India having the largest area of biotech crops [87,89]. In particular, India and China, the largest cotton producers in the world, grow 95% of the *Bt* cotton [89,90]. In terms of the number of approvals of GM events for food, feed, and cultivation, Japan is second, after the USA [89]. Japan is also one of the world’s biggest importers of GM crops, importing close to 100% of their corn and 94% of their oilseed supplies, which are largely soybean and canola [91]. In Europe, from 2016, only Spain and Portugal routinely cultivated an insect-resistant maize (MON810) [89]. In Africa, only a few of the 47 countries, including South Africa, Sudan, Kenya, Eswatini and, more recently, Malawi, Nigeria, and Ethiopia currently cultivate and market many GM crops. They also have many on-going field trials, usually supported by private foundations [87,92]. South Africa is the largest GM crop producer, having the ninth largest biotech crop area globally, and the first African country to have a regulatory framework for the cultivation, import, and export of GM crops [89].

In spite of the wide distribution of transgenic crops, it is noticeable that 99% of the transgenic crops are represented by four species, soybean (~48% of the global transgenic crop area), maize (~32%), cotton (~13%), and canola (~5%) [87]. Most of their products are not traditionally destined for human consumption; soybean crops provide soybean oil, mainly used as industrial adhesives, solvents, and lubricants, while bean meal is a high protein component in animal feed [93], and 55% of the total global production of maize is used as feed, 20% is used for other non-food uses such as ethanol production, and only 12% is used as food [94,95]. Other biotech crops are emerging, which account for 1% of the total cultivation area that ranges from 1.3 million hectares of alfalfa to less than 1000 hectares of squash, apples, and pineapple, including sugar beets, sugarcane, papaya, sunflower, potatoes, and eggplant [87]. In fact, further research is ongoing by private companies and public institutions that involves rice, banana, potato, wheat, chickpea, pigeon pea, and mustard with various economic and nutritional traits [87]. 

Since 1992, regulatory authorities have granted 4485 approvals for 403 GM events concerning 29 crops. Among these, 2115 approvals have been for food and 1514 approvals have been for feed, either for direct use or processing, while 856 approvals have been for environmental release and/or cultivation. The USA has had the highest number of GM events approved, followed by Japan, Canada, Brazil, and South Korea. Up-to-date information about the new approvals for biotech crops in many countries are in ISAAA [20]. 

Among the crops, maize has the greatest number of approved events, followed by cotton, potato, soybeans, and canola. The traits with the highest number of approvals in different countries include herbicide (mainly glyphosate and glufosinate) tolerant maize and soybean, insect-resistant maize, stacked herbicide-tolerant and insect-resistant maize, most of which have been obtained by private companies [20,87]. Of notable significance is the 6% increase in crops with stacked traits with insect resistance and herbicide tolerance, mainly approved in Brazil and in Argentina, demonstrating the greater interest for no or reduced tillage and reduced chemical use [87]. An interesting case is the biofortified Golden Rice (Golden Rice 2). While it is also registered as safe in Australia, USA, Canada, and New Zealand and possesses import approvals, the Philippines is the only country so far that has authorized its commercialization in 2021 and its direct use in food, feed, and processing, together with *Bt* eggplant [20,96].

With regard to GE-edited crops, the extent to which they can be developed, cultivated, and marketed will largely depend on how they are internationally regulated and whether they are subjected to the same costly law that applies to GMOs [97]. Despite GE potential, only a few genome-edited crop traits in soybean, canola, maize, mushroom, and camelina have been approved for commercialization to date. The first commercial crop generated using GE was the herbicide-tolerant variety of canola (FalcoTM Canola) developed by Cibus Global (Cibus Canola Event 5715), approved for commercial use in Canada in 2014 [98], and currently present in the Canadian and the USA markets [99]. Other GE crops with features designed to provide health benefits to consumers are already on the market. In USA and Canada, Calyxt markets the derivative product high-oleic soybean oil with healthier properties, longer shelf life, and better frying (CalynoTM) [87,100,101,102]. A nutritionally enhanced tomato, ”Sicilian Rouge High GABA” has been edited to contain increased levels of ɣ-aminobutyric acid (GABA) in the fruit, which has the properties of lowering blood pressure and promoting relaxation in consumers, and is marketed in Japan [103,104,105,106,107]. Currently, the common button mushroom (*Agaricus bisporus*) edited to have a reduced browning and spoilage [108] and th waxy corn with higher amylopectin content (Dupont-Pioneer’s) for use in the chemical industry [109], are also in the market in the USA. 

In 2022, the Argentine Biosafety Commission (Comisión Nacional de Biotecnología Agropecuaria or “CONABIA”) determined that three CRISPR genome-edited camelina lines produced by Yield10 do not contain any foreign-inserted DNA and are similar to conventionally bred camelina varieties; thus, they do not require any premarket authorizations. 

## 6. Global Regulation of Transgenic and Genome-Edited Crops

Differences in regulations among countries have major impacts on biotech crops’ research, global release, cultivation, trade both as import and export, and use as food and feed. Usually, many countries regulate the use and marketing of biotech crops based on the United Nations Cartagena Protocol on Biosafety, which defines “living modified organisms (LMO)” as those “possessing a novel combination of genetic material obtained through the use of modern biotechnology” [110]. This definition refers to transgenic technologies usually applied in the years before 2000, mainly based on the insertion of recombinant DNA in organisms beyond the species boundary, but it should be revised when considering new GE-edited crops and their products [111]. In fact, the genetic changes generated through GE, at least in their simplest form, are indistinguishable from those generated through natural or induced mutations, usually employed in mutation or conventional breeding programs, and the resulting genotypes are phenotypically indistinguishable. Furthermore, after self-crossing and selection cycles, in the edited plants, there is no longer any foreign DNA, and they only carry the desired mutations in the target gene(s). For this reason, the scientific community is now asking governments of countries in which GE-edited plants are considered to be low GM organisms, for a different regulation. 

Overall, release, commercialization, and the use of biotech crop regulations require different levels of evaluation. The end-use of such crops must first be considered. In fact, they are subjected to different regulatory frameworks, depending on whether the product is to be used as food and feed (regulatory framework for food and feed that must take into account potential risks to human and animal health), for cultivation purposes (regulatory framework for agriculture and/or the environment that considers environmental safety in a broad sense, use of pesticides, herbicides, increase in weeds, threats to biodiversity), or for industry [112]. Therefore, biotech crop approvals are time consuming and costly in many countries. Furthermore, since the costs of approval, subsequent management of crops, and continuous monitoring are very high, they are sustainable only by large multinational companies to the detriment of small companies and public research, leaving gaps in technological innovation and causing them to lose competitiveness.

Certain regulatory rules refer only to cultivation and not to the trade of GMO products, which is then regulated in a separate document and/or by a separate governmental body. In addition, several countries have rules regarding only the cultivation of GM crops for seed export but not for their use. Remarkably, some of these allow for the import of GM crop products as food and feed. 

A criterion that affects different regulations, mainly regarding GE, is one based on the process (technology used) or on the product obtained [62,113,114,115]. Process-oriented regulations consider all biotechnological technologies to be “novel” techniques compared to conventional breeding methods, and thus, they require specific legislation to be applied. Generally, in many countries, such as the EU or New Zealand, the GMO Directive refers to both the process used in genetic engineering and the product resulting from the use of such techniques [116], but it is interpreted as being based only on the process rather than the characteristics of the resulting product, despite the fact that, over 30 years of biosafety research, no generic risk caused by the technology itself has been demonstrated [117,118]. So far, Canada remains the only country that has based all its legislation on biotech crops, including GE-edited crops, on a product-oriented policy, regardless of the process used [119,120]. Albeit based on a case-by-case assessment, U.S. authorities have stated that when GE-edited plants contain no foreign DNA and the resultant change cannot be distinguished from a natural mutation, they do not constitute GMO [109] and, at present, only crops with multiple gene edits are regulated [121]. An updated description of regulatory approaches for GE in many countries has been provided by Schmidt et al. [122]. 

When legislations focus on the product, the most important factors for regulating GE-edited plants are the types of site-directed nuclease (SDN-1, SDN-2, and SDN-3) [111]. Over the last three years, more and more countries have changed their current regulations regarding plants with genome edits such as deletions (and small insertions) of one or several bases (SDN-1), which explicitly lack any introduced foreign sequences and are not distinguishable from natural occurring mutations or classically bred plants, exempting them from restrictions relate to GMO status [60,92,123,124,125]. Consequently, other than Canada and the USA, Japan, Israel, Australia, Brazil, Argentina, Chile, Paraguay, Ecuador, Columbia, Guatemala and Honduras, Philippines, Nigeria, and Kenya have issued their normative resolutions based on end-product and a case-by-case assessment and for most plants produced by SDN-1 (or SDN-2 once the CRISPR gene has been crossed out) it is sufficient to notify the competent authority that the plants are free of transgenes to exclude them from GMO legislation [60,62,87,91,126,127,128,129]. In particular, Australia and Japan have opted for a conservative threshold that allows them to regulate a crop edited using the SDN-2 technique as a GMO [130,131]. On the contrary, in the case of larger targeted insertions and modifications achievable via SDN-2 or SDN-3, easily detectable, further case-specific determinations would be required [132]. A noticeable policymaking discussion over proposals to treat SDN-1 as conventional new varieties is in progress in Nigeria, Kenya, and the UK [87]. After Brexit, following a recent consultation on the regulation of biotech technologies [133], the UK government introduced two legislative changes for GE crops [134] that exempted GM crops in which the final genome did not contain foreign DNA and with modifications which could have been obtained by conventional breeding, or which could have occurred naturally. Using the GMO regulation for field trials in England, regulatory measures were added to allow GE crops to be marketed. Several African countries are currently debating this as well. In India, the Department of Biotechnology of the Indian government has proposed GE guidelines that are based on a tiered regulatory approval process [135], whereas in the Philippines, Indonesia, and Bangladesh, discussions are ongoing [87]. 

Otherwise, in countries where the GE process is evaluated and not the product, the SDN type is irrelevant, and all these products are considered to be GMO according to old regulations; these countries include the EU, together with Sweden, Finland, Ireland, South Africa, and New Zealand [87,113,136]. Nevertheless, in the EU, the independent European Food Safety Authority (EFSA) considers SDN-1 to be a form of mutagenesis and proposes that different risk assessments of the final GE-edited plant product should only be considered if existing DNA is altered or if external DNA is inserted [137,138,139]. It is worthwhile to also mention the EFSA’s favorable opinion on the two other approaches belonging to the NBT, the intragenics/cisgenics, as effective strategies in leveraging genetic diversity and optimizing the crop genetic improvements without risk [140,141,142]. 

Among the non-EU countries, in Norway or Switzerland, but also in Russia, GE regulation is still in debate with open outcomes [92,143]. 

In the EU, the Directive 2001/18/EC, on the deliberate release into the environment of GMOs, defines a GMO as “an organism… in which the genetic material has been altered in a way that does not occur naturally by mating and/or natural recombination”, including in its regulation techniques involving recombinant nucleic acid (Article 2) (European Commission, 2001) [144]. The Directive regulates the cultivation and the market of GM crops in member states, based on a rigorous assessment of potential adverse effects on human health and the environment, limiting and, in many cases preventing, their use in agriculture and even research and experimentation in the field. It also allows member states to choose to “provisionally restrict or prohibit the use and/or sale of a GMO in all or in part of their territory”, if certain GM crops are approved for cultivation by the EU (Article 23). Together with the introduction of the safeguard clause in 2015 [145], this resulted in several EU countries having banned the cultivation of GM crops. In fact, in the EU in the last 25 years, only one event, an insect-resistant maize (MON810) has been routinely cultivated in Spain and Portugal [89]. The low cultivation rate of GM crops in the EU makes it heavily dependent on imports, particularly of GM soybean and maize for animal feed, from North and South America. 

The next Regulation (EC) No. 1829/2003, addressed to all 27 member states, specifically concerns GM food and feed produced from a GMO (Paragraph 16) and their imports [146] and, together with Regulation 1830/2003 regarding tracing and labeling of a GMO [147], they are aimed at ensuring that their authorization guarantees a high level of protection to human, animal, and environmental health (Article 1). In fact, the EU adheres to a precautionary principle, which enables, “where scientific data do not permit a complete evaluation of the risk, to stop distribution or order withdrawal from the market of products likely to be hazardous” [111]. As a result, the EU probably has the strictest regulations in the world for the presence of GMOs in food and feed with regard to human, animal, and environmental health; GMO safety; GMO thresholds; GMO labeling; GMO detection; and coexistence [92,111,146,147]. An extensive, case-by-case, science-based food and environmental safety evaluation of GMO food and feed is made by the independent European Food Safety Authority (EFSA). This creates many problems in cases of GE products whose mutations are indistinguishable from those arising spontaneously or through mutation breeding and cannot be easily detected and quantified in imported crops and products [120], affecting their traceability and labeling if they are exported from countries that do not regulate them. 

In July 2018, the Court of Justice of the European Union ruled that crop varieties resulting from the new techniques of site-directed mutagenesis are GMOs (Case C-528/16) [148] according to Article 2 of the “Cultivation Directive”, and they fall within the current legal framework. The High Court of France ruled that the crops obtained both through GE and through the classic “in vitro mutagenesis” are subject to the regulation on GMOs for which, in its territory, the cultivation or trade of such varieties is prohibited unless there is an approval under the GMO regulations [149]. This causes commercial problems, as some varieties will then be banned in France but not restricted in other member states. On 7 February 2023, the EU court released the C-688-21 law case’s adjudication results, stating that only organisms obtained by techniques or methods of mutagenesis conventionally used in various applications with a long tradition of safety are excluded from the scope of Directive 2001/18. This ruling is consistent with the conclusion of the EFSA report that mutants obtained by random mutagenesis in vivo and in vitro are indistinguishable from each other and they do not present any difference in terms of their safety, which has a long tradition. In fact, induced random mutagenesis, both in vivo and in vitro, was used in the selection of plant varieties well before 2001. Certainly, the adoption of the conclusions of this sentence is a positive fact and is in line with the recent openings of the EC towards edited crops.

On request of the Council of the European Union, the EC initiated a study regarding the status of “novel genomic techniques” under Union legislation, involving experts from member state competent authorities and stakeholders [150]. The results stated that “NGTs have the potential to contribute to a more sustainable food system as part of the objectives of the European Green Deal and the Farm to Fork Strategy”, and that the current GMO legislation is not suitable for these innovative technologies and their products [151]. The EC will now start a wide and open consultation process with all the interested stakeholders, including a public consultation, to discuss the possibility to design a new legal framework for these biotechnologies, which will be clear, evidence-based, and flexible enough to cope with future advances in science and technology [152].

## 7. Transgenic Versus GE Plants: What Possible Future?

Laws and national regulations represent the strongest limitation in development and adoption of genetically modified crops in certain countries. For this reason, in some countries such as those in the EU, the scientific community is trying to convince public opinion and the political class to accept that GE plants are identical to lines obtained through classical mutagenesis and breeding. On the one hand, this effort is, for sure, an appreciable compromise to have the possibility of using the most advanced biotechnological approaches to genetically improve crops. On the other hand, we wonder if GE alone can provide valid answers to all the questions important for crop breeding. The current advancement in transgenesis and GE technologies enable many applications that are suitable for improving crop yield and quality, food security, disease resistance, and the ability to cope with climate change. The techniques both have advantages and disadvantages which condition the choice of the strategy to be adopted according to the character to be improved. Indeed, transgenesis offers the chance of introducing into a crop a useful gene from virtually every species to modify any trait of choice. Traits such as resistance to herbicides or insects are already easily and widely obtained through transgenesis, but the resulting biotech crops are subject to multiple regulatory restrictions in many countries and have negative public acceptance. Nevertheless, it would be very difficult to obtain crops that are resistant to herbicides by acting on plants through editing of their own genes. In fact, although the use of CRISPR/Cas technology for precise modifications of genes related to herbicide resistance through different pathway repairs has been recently applied, mainly targeting genes such as *acetolactate synthase* (*ALS*), *acetyl-CoA carboxylase* (*ACCase*), and *5-enolpyruvylshikimate-3-phosphate synthase (EPSPS)* [153] has several issues that remain to be addressed. One of the primary challenges is to find other potential genes for creating herbicide-resistant crops by GE and to understand which pathway they act on more appropriately. Currently, there are still limited studies related to their identification. Another trait for which significant success has been obtained through transgenesis is insect resistance. Recently, soybean resistance to leaf-chewing insects, *H. armigera* and *S. litura*, was obtained both by overexpressing the *GmUGT* gene and by CRISPR/Cas9-mediated targeted mutagenesis of the *GmUGT* genes [154] that altered the flavonoid content and expression patterns of the genes related to flavonoid biosynthesis and the defense response. Mutant and overexpressing lines both exhibited no obvious phenotypic changes and must also be subjected to field trials to evaluate the effectiveness and stability of the new phenotype with regard to the general performance of the plant. Nevertheless, GE cannot replace transgenesis when the new desired trait requires genes present in different species, even sexually incompatible, such as the example of golden rice which was made capable of producing vitamin A. On the contrary, studies with field trials have revealed very promising results for GE to increase grain yield, a trait which has not been targeted on a large scale through transgenesis. Biotic and abiotic stress resistance, increased uptake of nutrient compounds, and reduced toxic compounds are targets of the GE approach and, together with increased yield, have the advantage of a reduced degree of regulatory oversight [96,155,156].

To be effective, regulatory biotechnology should be accompanied by significant public and private investments in research Biotechnologies are not always the only solution for genetic improvement of all the traits of interest and for all the crops, nor are they always easier and practicable. To have a consistent impact on the agricultural system as a whole, therefore, these investments should not only concern biotechnological approaches in the narrow sense, but should be extended to the entire area of genomics, considering all the advanced technologies necessary to increase the efficiency of genetic improvement (genomic assisted selection). With this goal, it would be possible to meet the true needs of modern agriculture, which are those of selected new varieties and hybrids. 

Regarding productive, sustainable, and competitive agriculture, the contributions of advanced plant genetics are essential, and strong intervention by the scientific community on political decision-makers is necessary. However, to maintain trust in science, it is essential to make greater efforts to ensure traceability and labeling of biotech crops and their products. It is also important to promote a broad dialogue with relevant stakeholders and the public at large, and to provide robust and independent evidence in a systematic and transparent way, especially when reasons other than scientific ones, such as those based on ethical, legal, social, and economic considerations, heavily influence the decision-making process. In addition, trust in governments and regulatory authorities to properly legislate these technologies ensuring environmental and health safety standards is a key component of public acceptance.

## 8. Conclusions and Future Perspectives

In this review, we focused on recent studies that have tested modified lines, in the field, with good results for different agronomic traits of interest. These lines can be considered to be the most promising materials available and as candidates for approval and marketing in the near future. In addition, we must recognize the importance of more basic studies in which potential targets to develop improved crops are investigated, such as pathogenicity determinants in the case of resistance against different pathogens. For example, it could be possible to promote plant resistance to geminivirus betasatellites, reducing damage at the level of chloroplasts caused by its pathogenicity determinant [157].

In conclusion, to adapt national agriculture to the future and to maintain the competitiveness of the national agricultural sector in response to climate change and the objectives of modern challenges, it would be strategic to promote, in each country, a coordinated public–private genetic improvement system based on the most advanced genomic technologies. As regulatory, commercial, and intellectual property frameworks are continually being debated and decided upon, there is an urgent need for policy and civil makers to take timely action to ensure that the best that science can offer contributes to effectively improving agriculture globally.

## Data Availability

Not applicable.

## Supplementary Materials

The supporting information can be downloaded at: https://www.mdpi.com/article/10.3390/ijms24087122/s1.

## References

[1] Collard B.C., Mackill D.J. (2008). Marker-assisted selection: An approach for precision plant breeding in the twenty-first century. Philos. Trans. R. Soc. Lond. B Biol. Sci..

[2] Sikora P., Chawade A., Larsson M., Olsson J., Olsson O. (2011). Mutagenesis as a tool in plant genetics, functional genomics, and breeding. Int. J. Plant Genom..

[3] McCallum C.M., Comai L., Greene E.A., Henikoff S. (2000). Targeted screening for induced mutations. Nat. Biotechnol..

[4] Kamthan A., Chaudhuri A., Kamthan M., Datta A. (2016). Genetically modified (GM) crops: Milestones and new advances in crop improvement. Theor. Appl. Genet..

[5] Kamburova V.S., Nikitina E.V., Shermatov S.E., Buriev Z.T., Kumpatla S.P., Emani C., Abdurakhmonov I.Y. (2017). Genome Editing in Plants: An Overview of Tools and Applications. Int. J. Agron..

[6] Chen K., Gao C. (2014). Targeted Genome Modification Technologies and Their Applications in Crop Improvements. Plant Cell Rep..

[7] Puchta H. (2017). Applying CRISPR/Cas for Genome Engineering in Plants: The Best is Yet to Come. Curr. Opin. Plant Biol..

[8] Ma X., Zhang Q., Zhu Q., Liu W., Chen Y., Qiu R., Wang B., Yang Z., Li H., Lin Y. (2015). A robust CRISPR/Cas9 System for Convenient, High-efficiency Multiplex Genome Editing in Monocot and Dicot Plants. Mol. Plant.

[9] Yin K., Gao C., Qiu J.-L. (2017). Progress and prospects in plant genome editing. Nat. Plants.

[10] Brookes G., Barfoot P. (2020). GM Crop Technology Use 1996–2018: Farm Income and Production Impacts. GM Crops Food.

[11] Ricroch A.E., Martin-Laffon J., Rault B., Pallares V.C., Kuntz M. (2022). Next biotechnological plants for addressing global challenges: The contribution of transgenesis and new breeding techniques. New Biotechnol..

[12] Dill G.M. (2005). Glyphosate-resistant crops: History, status and future. Pest Manag. Sci..

[13] Pagano M., Miransari M., Miransari M. (2016). The importance of soybean production worldwide. Abiotic and Biotic Stresses in Soybean Production.

[14] Xu H., Guo Y., Qiu L., Ran Y. (2022). Progress in Soybean Genetic Transformation Over the Last Decade. Front. Plant Sci..

[15] Sripaoraya S., Keawsompong S., Insupa P., Power J.B., Davey M.R., Srinives P. (2006). Genetically manipulated pineapple: Transgene stability, gene expression and herbicide tolerance under field conditions. Plant Breed..

[16] Wang Y., Wisniewski M., Meilan R., Cui M., Fuchigami L. (2006). Transgenic tomato (*Lycopersicon esculentum*) overexpressing cAPX exhibits enhanced tolerance to UV-B and heat stress. J. Appl. Hortic..

[17] Jiang J., Bai J., Li S., Xiaorong L., Liyong Y., Yuke H. (2018). HTT2 promotes plant thermotolerance in *Brassica rapa*. BMC Plant Biol..

[18] Chen L., Yang H., Fang Y., Guo W., Chen H., Zhang X., Dai W., Chen S., Hao Q., Yuan S. (2021). Overexpression of GmMYB14 improves high-density yield and drought tolerance of soybean through regulating plant architecture mediated by the brassinosteroid pathway. Plant Biotechnol. J..

[19] González F.G., Capella M., Ribichich K.F., Curín F., Giacomelli J.I., Ayala F., Watson G., Otegui M.E., Chan R.L. (2019). Field-grown transgenic wheat expressing the sunflower gene HaHB4 significantly outyields the wild type. J. Exp. Bot..

[20] ISAAA GM Approval Database. http://www.isaaa.org/gmapprovaldatabase/default.asp.

[21] Anwar A., Kim J.K. (2020). Transgenic Breeding Approaches for Improving Abiotic Stress Tolerance: Recent Progress and Future Perspectives. Int. J. Mol. Sci..

[22] Mores A., Borrelli G.M., Laidò G., Petruzzino G., Pecchioni N., Amoroso L.G.M., Desiderio F., Mazzucotelli E., Mastrangelo A.M., Marone D. (2021). Genomic Approaches to Identify Molecular Bases of Crop Resistance to Diseases and to Develop Future Breeding Strategies. Int. J. Mol. Sci..

[23] Xiangdong W., Congyu L., Zhijing L., Changming Y. (2007). Analysis on virus resistance and fruit quality for T4 generation of transgenic papaya. Front. Biol. China.

[24] Dutt M., Barthe G., Irey M., Grosser J. (2015). Transgenic Citrus Expressing an Arabidopsis NPR1 Gene Exhibit Enhanced Resistance against Huanglongbing (HLB.; Citrus Greening). PLoS ONE.

[25] Tu J., Datta K., Khush G.S., Zhang Q., Datta S.K. (2000). Field performance of Xa21 transgenic indica rice (*Oryza sativa* L.), IR72. Theor. Appl. Genet..

[26] Dastan S., Ghareyazie B., Abdollahi S. (2020). Field trial evidence of non-transgenic and transgenic Bt. rice genotypes in north of Iran. J. Genet. Eng. Biotechnol..

[27] Budeguer F., Enrique R., Perera M.F., Racedo J., Castagnaro A.P., Noguera A.S., Welin B. (2021). Genetic Transformation of Sugarcane, Current Status and Future Prospects. Front. Plant Sci..

[28] Wang W.Z., Yang B.P., Feng X.Y., Cao Z.Y., Feng C.L., Wang J.G., Xiong G.R., Shen L.B., Zeng J., Zhao T.T. (2017). Development and Characterization of Transgenic Sugarcane with Insect Resistance and Herbicide Tolerance. Front. Plant Sci..

[29] Gilbert R.A., Gallo-Meagher M., Comstock J.C., Miller J.D., Jain M., Abouzid A. (2005). Agronomic evaluation of sugarcane lines transformed for resistance to strain E. Crop Sci..

[30] Gilbert R.A., Glynn N.C., Comstock J.C., Davis M.J. (2009). Agronomic performance and genetic characterization of sugarcane transformed for resistance to sugarcane yellow leaf virus. Field Crop Res..

[31] Yao W., Ruan M., Qin L., Yang C., Chen R., Chen B., Zhang M. (2017). Field Performance of Transgenic Sugarcane Lines Resistant to Sugarcane Mosaic Virus. Front Plant Sci..

[32] Jones J.D., Witek K., Verweij W., Jupe F., Cooke D., Dorling S., Tomlinson L., Smoker M., Perkins S., Foster S. (2014). Elevating crop disease resistance with cloned genes. Philos. Trans. R. Soc. Lond. B Biol. Sci..

[33] Ghislain M., Byarugaba A.A., Magembe E., Njoroge A., Rivera C., Román M.L., Tovar J.C., Gamboa S., Forbes G.A., Kreuze J.F. (2019). Stacking three late blight resistance genes from wild species directly into African highland potato varieties confers complete field resistance to local blight races. Plant Biotechnol. J..

[34] Webi E.N., Kariuki D., Kinyua J., Njoroge A., Ghislain M., Magembe E. (2019). Extreme resistance to late blight disease by transferring 3 *R* genes from wild relatives into African farmer-preferred potato varieties. Afr. J. Biotechnol..

[35] Yang J., Xing G., Niu L., He H., Guo D., Du Q., Qian X., Yao Y., Li H., Zhong X. (2018). Improved oil quality in transgenic soybean seeds by RNAi-mediated knockdown of GmFAD2-1B. Transgenic Res..

[36] Park S., Kim S.H., Park S., Lee H., Lee J.S., Park W.S., Ahn M., Kim Y., Jeong J.C., Lee H. (2015). Enhanced accumulation of carotenoids in sweet potato plants overexpressing IbOr-Ins gene in purple-fleshed sweet potato cultivar. Plant Physiol. Biochem..

[37] Frauenlob J., Scharl M., Stefano D’Amico S., Regine Schoenlechner R. (2018). Effect of different lipases on bread staling in comparison with Diacetyl tartaric ester of monoglycerides (DATEM). Cereal Chem..

[38] Larkin P.J., Liu Q., Vanhercke T., Zhou X.R., Bose U., Broadbent J.A., Colgrave M.L., Ral J.P., Reynolds K.B., Sun M. (2021). Transgenic wheat with increased endosperm lipid—Impacts on grain composition and baking quality. J. Cereal Sci..

[39] Taylor D.C., Zhang Y., Kumar A., Francis T., Giblin E.M., Barton D.L., Ferrie J.R., Laroche A., Shah S., Zhu W. (2009). Molecular modification of triacylglycerol accumulation by over-expression of DGAT1 to produce canola with increased seed oil content under field conditions. Botany.

[40] Ramireddy E., Hosseini S.A., Eggert K., Gillandt S., Gnad H., von Wirén N., Schmülling T. (2018). Root Engineering in Barley: Increasing Cytokinin Degradation Produces a Larger Root System, Mineral Enrichment in the Shoot and Improved Drought Tolerance. Plant Physiol..

[41] James C.M., Krattiger A.F. (1996). The First Decade of Crop Biotechnology. Global Review of the Field Testing and Commercialization of Transgenic Plants, 1986 to 1995.

[42] Kramer M.G., Redenbaugh K. (1994). Commercialization of a tomato with an antisense polygalacturonase gene: The FLAVR SAVR™ tomato story. Euphytica.

[43] Schouten H.J., Krens F.A., Jacobsen E. (2006). Cisgenic Plants are Similar to Traditionally Bred Plants: International Regulations for Genetically Modified Organisms Should be Altered to Exempt Cisgenesis. EMBO Rep..

[44] Espinoza C., Schlechter R., Herrera D., Torres E., Serrano A., Medina C., Arce-Johnson P. (2013). Cisgenesis and Intragenesis: New Tools for Improving Crops. Biol. Res..

[45] Holme I.B., Wendt T., Holme P.B. (2013). Intragenesis and cisgenesis as alternatives to transgenic crop development. Plant Biotechnol. J..

[46] Cardi T. (2016). Cisgenesis and genome editing: Combining concepts and effortsfor a smarter use of genetic resources in crop breeding. Plant Breed..

[47] Moradpour M., Abdullah S.N.A., Abdullah S., Chai-Ling H., Wagstaff C. (2017). Cisgenesis and Intragenesis as New Strategies for Crop Improvement. Crop Improvement: Sustainability Through Leading-Edge Technology.

[48] Gadaleta A., Giancaspro A., Blechl A.E., Blanco A. (2008). A transgenic durum wheat line that is free of marker genes and expresses *1DY10*. J. Cereal Sci..

[49] Maltseva E., Ismagul A., Iskakova G., Chirkin A., Skiba Y., Ismagulova G., Eliby S., Aitkhozhinaet N. (2014). Wheat Cisgenic Transformation with Class I *Chitinase* Gene. J. Biotechnol..

[50] Holme I.B., Dionisio G., Brinch-Pedersen H., Wendt T., Madsen C.K., Vincze E., Holm P.B. (2012). Cisgenic barley with improved phytase activity. Plant Biotechnol. J..

[51] Singh V., Singh S., Shikha K., Kumar A. (2018). Cisgenesis a Sustainable Approach of Gene Introgression and Its Utilization in Horticultural Crops: A Review. Int. J. Curr. Microbiol. Appl. Sci..

[52] Haverkort A.J., Boonekamp P.M., Hutten R., Jacobsen E., Lotz L.A.P., Kessel G.J.T., Vossen J.H., Visser R.G.F. (2016). Durable Late Blight Resistance in Potato through Dynamic Varieties Obtained by Cisgenesis: Scientific and Societal Advances in the DuRPh Project. Potato Res..

[53] Haesaert G., Vossen J.H., Custers R., De Loose M., Haverkort A., Heremans B., Hutten R., Kessel G., Landschoot S., Van Droogenbroeck B. (2015). Transformation of the potato variety Desiree with single or multiple resistance genes increases resistance to late blight under field conditions. Crop Prot..

[54] Krause S.M.B., Näther A., Ortiz Cortes V., Mullins E., Kessel G.J.T., Lotz L.A.P., Tebbe C.C. (2020). No Tangible Effects of Field-Grown Cisgenic Potatoes on Soil Microbial Communities. Front. Bioeng. Biotechnol..

[55] Chawla R., Shakya R., Rommens C.M. (2012). Tuber-specific silencing of asparagine synthetase-1 reduces the acrylamide-forming potential of potatoes grown in the field without affecting tuber shape and yield. Plant Biotechnol. J..

[56] de Vetten N., Wolters A., Raemakers K., van der Meer I., ter Stege R., Heeres E., Heeres P., Visser R. (2003). A transformation method for obtaining marker-free plants of a cross-pollinating and vegetatively propagated crop. Nat. Biotech..

[57] Joshi S.G., Schaart J.G., Groenwold R., Jacobsen E., Schouten H.J., Krens F.A. (2011). Functional analysis and expression profiling of *HcrVf1* and *HcrVf2* for development of scab resistant cisgenic and intragenic apples. Plant Mol. Biol..

[58] Vanblaere T., Szankowski I., Schaart J., Schouten H., Flachowsky H., Broggini G.A.L., Gessler C. (2011). The development of a cisgenic apple plant. J. Biotechnol..

[59] Hamdan M.F., Karlson C.K.S., Teoh E.Y., Lau S.-E., Tan B.C. (2022). Genome Editing for Sustainable Crop Improvement and Mitigation of Biotic and Abiotic Stresses. Plants.

[60] Menz J., Modrzejewski D., Hartung F., Wilhelm R., Sprink T. (2020). Genome Edited Crops Touch the Market: A View on the Global Development and Regulatory Environment. Front. Plant Sci..

[61] Sedeek K.E.M., Mahas A., Mahfouz M. (2019). Plant Genome Engineering for Targeted Improvement of Crop Traits. Front. Plant Sci..

[62] Metje-Sprink J., Sprink T., Hartung F. (2020). Genome-edited plants in the field. Curr. Opin. Biotechnol..

[63] Li M., Li X., Zhou Z., Wu P., Fang M., Pan X., Lin Q., Luo W., Wu G., Li H. (2016). Reassessment of the four yield-related genes Gn1a, DEP1, GS3, and IPA1 in rice using a CRISPR/Cas9 system. Front Plant Sci..

[64] Shen L., Hua Y., Fu Y., Li J., Liu Q., Jiao X., Xin G., Wang J., Wang X., Yan C. (2017). Rapid generation of genetic diversity by multiplex CRISPR/Cas9 genome editing in rice. Sci. China Life Sci..

[65] Zhou J., Xin X., He Y., Chen H., Li Q., Tang X., Zhong Z., Deng K., Zheng X., Akher S.A. (2019). Multiplex QTL editing of grain-related genes improves yield in elite rice varieties. Plant Cell Rep..

[66] Hu X., Cui Y., Dong G., Feng A., Wang D., Zhao C., Zhang Y., Hu J., Zeng D., Guo L. (2019). Using CRISPR-Cas9 to generate semi-dwarf rice lines in elite landraces. Sci. Rep..

[67] Zhang J., Zhang H., Li S., Li J., Yan L., Xia L. (2021). Increasing yield potential through manipulating of an ARE1 ortholog related to nitrogen use efficiency in wheat by CRISPR/Cas9. J. Integr. Plant Biol..

[68] Shi J., Gao H., Wang H., Lafitte H.R., Archibald R.L., Yang M., Hakimi S.M., Mo H., Habben J.E. (2017). ARGOS 8 variants generated by CRISPR-Cas9 improve maize grain yield under field drought stress conditions. Plant Biotechnol. J..

[69] Zhang A., Liu Y., Wang F., Li T., Chen Z., Kong D., Bi J., Zhang F., Luo X., Wang J. (2019). Enhanced rice salinity tolerance via CRISPR/Cas9-targeted mutagenesis of the OsRR22 gene. Mol. Breed..

[70] Takagi H., Tamiru M., Abe A., Yoshida K., Uemura A., Yaegashi H., Obara T., Oikawa K., Utsushi H., Kanzaki E. (2015). MutMap accelerates breeding of a salt-tolerant rice cultivar. Nat. Biotechnol..

[71] Zeng X., Luo Y., Vu N.T.Q., Shen S., Xia K., Zhang M. (2020). CRISPR/Cas9-mediated mutation of OsSWEET14 in rice cv. Zhonghua11 confers resistance to Xanthomonas oryzae pv. oryzae without yield penalty. BMC Plant Biol..

[72] Zhou Y., Xu S., Jiang N., Zhao X., Bai Z., Liu J., Yao W., Tang W., Xiao G., Chao Lv C. (2022). Engineering of rice varieties with enhanced resistances to both blast and bacterial blight diseases via CRISPR/Cas9. Plant Biotech. J..

[73] Wang F., Wang C., Liu P., Lei C., Hao W., Gao Y., Liu Y.G., Zhao K. (2016). Enhanced Rice Blast Resistance by CRISPR/Cas9-Targeted Mutagenesis of the ERF Transcription Factor Gene OsERF922. PLoS ONE.

[74] Gao H., Gadlage M.J., Lafitte H.R., Lenderts B., Yang M., Schroder M., Farrell J., Snopek K., Peterson D., Feigenbutz L. (2020). Superior field performance of waxy corn engineered using CRISPR-Cas9. Nat. Biotechnol..

[75] Tang L., Mao B., Li Y., Lv Q., Zhang L., Chen C., He H., Wang W., Zeng X., Shao Y. (2017). Knockout of OsNramp5 using the CRISPR/Cas9 system produces low Cd-accumulating indica rice without compromising yield. Sci. Rep..

[76] Yang C.H., Zhang Y., Huang C.F. (2019). Reduction in cadmium accumulation in japonica rice grains by CRISPR/Cas9-mediated editing of OsNRAMP5. J. Integr. Agric..

[77] Songmei L., Jie J., Yang L., Jun M., Shouling X., Yuanyuan T., Youfa L., Qingyao S., Jianzhong H. (2019). Characterization and evaluation of OsLCT1 and OsNramp5 mutants generated through CRISPR/Cas9-mediated mutagenesis for breeding low Cd rice. Rice Sci..

[78] Nieves-Cordones M., Mohamed S., Tanoi K., Kobayashi N.I., Takagi K., Vernet A., Guiderdoni E., Périn C., Sentenac H., Véry A.A. (2017). Production of low-Cs+ rice plants by inactivation of the K+ transporter OsHAK1 with the CRISPR-Cas system. Plant J..

[79] Neequaye M., Stavnstrup S., Harwood W., Lawrenson T., Hundleby P., Irwin J., Troncoso-Rey P., Saha S., Traka M.H., Mithen R. (2021). CRISPR-Cas9-Mediated Gene Editing of *MYB28* Genes Impair Glucoraphanin Accumulation of Brassica oleracea in the Field. CRISPR J..

[80] Raffan S., Sparks C., Huttly A., Hyde L., Martignago D., Mead A., Hanley S.J., Wilkinson P.A., Barker G., Edwards K.J. (2021). Wheat with Greatly Reduced Accumulation of Free Asparagine in the Grain, Produced by CRISPR/Cas9 Editing of Asparagine Synthetase Gene TaASN2. Plant Biotechnol. J..

[81] Faure J.D., Napier J.A. (2018). Europe’s first and last field trial of gene-edited plants?. Elife.

[82] Salie M.J., Zhang N., Lancikova V., Xu D., Thelen J.J. (2016). A Family of Negative Regulators Targets the Committed Step of de Novo Fatty Acid Biosynthesis. Plant Cell.

[83] Yield10 Bioscience, Inc. Yield10 Bioscience Field Test Results Show Seed Oil Content Increase in Camelina and Canola. https://www.yield10bio.com/press/yield10-bioscience-field-test-results-show-seed-oil-content-increase-in-camelina-and-canola.

[84] Knisley S. 2021 Gene Editing Innovations Present many Benefits to Farmers and Their Customers. https://www.uswheat.org/wheatletter/gene-editing-innovationspresent-many-benefits-to-farmers-and-their-customers.

[85] Meng X., Yu H., Zhang Y., Zhuang F., Song X., Gao S., Gao C., Li J. (2017). Construction of a Genome-Wide Mutant Library in Rice Using CRISPR/Cas9. Mol. Plant..

[86] Dhariwal G.K., Laroche A. (2017). The future of genetically engineered plants to stabilize yield and improve feed. Anim. Front..

[87] ISAAA (2019). Global Status of Commercialized Biotech/GM Crops in 2019 (ISAAA Brief No. 55).

[88] Report Linker 2023 Global Agricultural Biotechnology Industry: Global Agricultural Biotechnology Market to Reach $88.9 Billion by 2030. https://www.reportlinker.com/p04838495/Global-Agricultural-Biotechnology-Biotechnology.html.

[89] ISAAA (2018). Global Status of Commercialized Biotech/GM Crops in 2018: Biotech Crops Continue to Help Meet the Challenges of Increased Population and Climate Change (ISAAA Brief N. 54).

[90] Shahbandeh M. (2020). World Cotton Production by Country 2019/2020.

[91] USDA Foreign Agricultural Service (USDA FAS) Agricultural Biotechnology Annual—Japan. GAIN Report Number: JA2021-0140. https://www.fas.usda.gov/data/japan-agricultural-biotechnology-annual-3.

[92] Turnbull C., Lillemo M., Hvoslef-Eide T.A.K. (2021). Global Regulation of Genetically Modified Crops Amid the Gene Edited Crop Boom—A Review. Front. Plant Sci..

[93] Nosowitz D. 2017 Soy Is Set to Become Our Biggest Crop by Acreage. But What Are We Doing with This Soy?. https://modernfarmer.com/2017/12/soy-set-become-biggest-crop-acreage-soy/.

[94] Ranum P., Peña-Rosas J.P., Garcia-Casal M.N. (2014). Global Maize Production, Utilization, and Consumption. Ann. N. Y. Acad. Sci..

[95] FAO New Food Balances. FAOSTAT. http://www.fao.org/faostat/en/#data/FBS.

[96] Hamdan M.F., Mohd Noor S.N., Abd-Aziz N., Pua T.-L., Tan B.C. (2022). Green Revolution to Gene Revolution: Technological Advances in Agriculture to Feed the World. Plants.

[97] Jenkins D., Dobert R., Atanassova A., Pavely C. (2021). Impacts of the Regulatory Environment for Gene Editing on Delivering Beneficial Products. In Vitro Cell Dev. Biol. Plant..

[98] Cibus Cibus Global Announces Approval of First Commercial Product SU Canola in Canada 2014. http://cibus.com/press/press031814.php.

[99] Cibus Canada Inc. Marketed Products. https://www.cibus.com/marketed-products.php.

[100] Demorest Z.L., Coffman A., Baltes N.J., Stoddard T.J., Clasen B.M., Luo S., Retterath A., Yabandith A., Gamo M.E., Bissen J. (2016). Direct Stacking of Sequence-specific Nuclease-induced Mutations to Produce High Oleic and Low Linolenic Soybean Oil. BMC Plant Biol..

[101] Calyxt Inc (2015). Calyxt Launches Field Trials of High Oleic Soybean.

[102] Calyxt Inc (2019). First Commercial Sale of Calyxt High Oleic Soybean Oil on the U.S. Market.

[103] Nonaka S., Arai C., Takayama M., Matsukura C., Ezura H. (2017). Efficient Increase of ɣ-aminobutyric Acid (GABA) Content in Tomato Fruits by Targeted Mutagenesis. Sci. Rep..

[104] USDA Foreign Agricultural Service (USDA FAS) Japan: Japan Determines Genome Edited Tomato Will Not be Regulated as GE. https://www.fas.usda.gov/data/japan-japan-determines-genome-edited-tomato-will-not-be-regulated-ge.

[105] Sanatech Seed First Genome Edited Tomato with Increased GABA In the World. https://sanatech-seed.com/en/20201211-1-2/.

[106] Sanatech Seed Launch of Genome Edited Tomato Fruit for Purchase. https://sanatech-seed.com/en/20210915-2/.

[107] Waltz E. (2021). GABA-enriched Tomato is First CRISPR-Edited Food to Enter Market. Nat. Biotech..

[108] Waltz E. (2016). Gene-Edited CRISPR Mushroom Escapes US Regulation. Nature.

[109] USDA Animal and Plant Health Inspection Service (USDA APHIS) Regulated Article Letters of Inquiry. https://www.aphis.usda.gov/aphis/ourfocus/biotechnology/am-i-regulated/Regulated_Article_Letters_of_Inquiry.

[110] Secretariat of the Convention on Biological Diversity Cartagena Protocol on Biosafety to the Convention on Biological Diversity: Text and annexes. Montreal: Secretariat of the Convention on Biological Diversity. https://bch.cbd.int/protocol/text/.

[111] Wolt J.D., Wang K., Yang B. (2016). The Regulatory Status of Genome-edited Crops. Plant Biotechnol. J..

[112] Huesing J.E., Andres D., Braverman M.P., Burns A., Felsot A.S., Harrigan G.G., Hellmich R.L., Reynolds A., Shelton A.M., Jansen van Rijssen W. (2016). Global Adoption of Genetically Modified (GM) Crops: Challenges for the Public Sector. J. Agric. Food Chem..

[113] Sprink T., Eriksson D., Schiemann J., Hartung F. (2016). Regulatory Hurdles for Genome Editing: Process-vs. Product-Based Approaches in Different Regulatory Contexts. Plant Cell Rep..

[114] Medvedieva M.O., Blume Y.B. (2018). Legal Regulation of Plant Genome Editing with the CRISPR/Cas9 Technology as an Example. Cytol. Genet..

[115] Eckerstorfer M.F., Engelhard M., Heissenberger A., Simon S., Teichmann H. (2019). Plants developed by new genetic modification techniques—Comparison of existing regulatory frameworks in the EU and Non-EU countries. Front. Bioeng. Biotechnol..

[116] Abbott A. (2015). Europe’s Genetically Edited Plants Stuck in Legal Limbo. Nature.

[117] Nicolia A., Manzo A., Veronesi F., Rosellini D. (2014). An overview of the last 10 years of genetically engineered crop safety research. Crit. Rev. Biotechnol..

[118] Leopoldina D.F.G., Akademieunion (2019). Wege zu Einer Wissenschaftlich Begründeten, Differenzierten Regulierung Genomeditierter Planzen in der EU: Stellungnahme = Towards a Scientifically Justified, differentiated Regulation of Genome Edited Plants in the EU. Halle: Deutsche Akademie der Naturforscher.

[119] Smyth S.J. (2017). Canadian Regulatory Perspectives on Genome Engineered Crops. GM Crops Food.

[120] Ellens K.W., Levac D., Pearson C., Savoie A., Strand N., Louter J., Tibelius C. (2019). Canadian Regulatory Aspects of Gene Editing Technologies. Transgenic Research.

[121] Hoffman N.E. (2021). Revisions to USDA Biotechnology Regulations: The SECURE Rule. Proc. Natl. Acad. Sci. USA.

[122] Schmidt S.M., Belisle M., Frommer W.B. (2020). The Evolving Landscape Around Genome Editing in Agriculture: Many Countries have Exempted or Move to Exempt Forms of Genome Editing from GMO Regulation of Crop Plants. EMBO Rep..

[123] Grohmann L., Keilwagen J., Duensing N., Dagand E., Hartung F., Wilhelm R., Bendiek J., Sprink T. (2019). Detection and Identification of Genome Editing in Plants: Challenges and Opportunities. Front. Plant Sci..

[124] Whelan A.I., Gutti P., Lema M.A. (2020). Gene Editing Regulation and Innovation Economics. Front. Bioeng. Biotechnol..

[125] Entine J., Felipe M.S.S., Groenewald J.H., Kershen D.L., Lema M., McHughen A., Nepomuceno A.L., Ohsawa R., Ordonio R.L., Parrott W.A. (2021). Regulatory Approaches for Genome Edited Agricultural Plants in Select Countries and Jurisdictions Around the World. Transgenic Res..

[126] Lema M.A. (2019). Regulatory Aspects of Gene Editing in Argentina. Transgenic Res..

[127] USDA Foreign Agricultural Service (USDA FAS) (2018). Israel Agricultural Biotechnology Annual 2018. GAIN Report Number: IS18011. https://www.fas.usda.gov/data/israel-agricultural-biotechnology-annual-2.

[128] USDA Foreign Agricultural Service (USDA FAS) (2018). Japan Discusses Genome Editing Technology. GAIN Report Number: JA8048. https://www.fas.usda.gov/data/japan-japan-discusses-genome-editing-technology.

[129] Gatica-Arias A. (2020). The Regulatory Current Status of Plant Breeding Technologies in Some Latin American and the Caribbean Countries. Plant Cell. Tissue Organ Cult..

[130] Thygesen P. (2019). Clarifying the Regulation of Genome Editing in Australia: Situation for Genetically Modified Organisms. Transgenic Res..

[131] Tsuda M., Watanabe K.N., Ohsawa R. (2019). Regulatory Status of Genome edited Organisms Under the Japanese Cartagena Act. Front. Bioeng. Biotechnol..

[132] Camacho A., Van Deynze A., Chi-Ham C., Bennett A.B. (2014). Genetically Engineered Crops that Fly Under the US Regulatory Radar. Nat. Biotechnol..

[133] Department for Environment Food & Rural Affairs (DEFRA) The Regulation of Genetic Technologies—A Public Consultation on the Regulation of Genetic Technologies. www.gov.uk/government/publications.

[134] Department for Environment Food & Rural Affairs (DEFRA) Genetic Technologies Regulation: Government Response. https://www.gov.uk/government/consultations/genetic-technologies-regulation/outcome/genetic-technologies-regulation-government-response.

[135] Fernandes V. India’s Genome-Editing Draft Guidelines are Needlessly Restrictive. 2020, India: Smart Indian Agriculture. https://smartindianagriculture.com/indias-genome-editing-draft-guidelines-are-needlessly-restrictive/.

[136] van der Meer P., Angenon G., Bergmans H., Buhk H.-J., Callebaut S., Chamon M., Eriksson D., Gheysen G., Harwood W., Hundleby P. (2021). The Status Under EU Law of Organisms Developed Through Novel Genomic Techniques. Eur. J. Risk Regul..

[137] EFSA GMO Panel (2012). Scientific opinion addressing the safety assessment of plants developed using Zinc Finger Nuclease 3 and other Site-Directed Nucleases with similar function. EFSA J..

[138] EFSA GMO Panel (2020). Applicability of the EFSA Opinion on site-directed nucleases type 3 for the safety assessment of plants developed using site-directed nucleases type 1 and 2 and oligonucleotide-directed mutagenesis. EFSA J..

[139] Paraskevopoulos K., Federici S. (2021). Overview of EFSA and European national authorities’ scientific opinions on the risk assessment of plants developed through New Genomic Techniques. EFSA J..

[140] EFSA GMO Panel (2012). Scientific opinion addressing the safety assessment of plants developed through cisgenesis and intragenesis. EFSA J..

[141] EFSA GMO Panel (2022). Updated scientific opinion on plants developed through cisgenesis and intragenesis. EFSA J..

[142] EFSA GMO Panel (2022). Statement on criteria for risk assessment of plants produced by targeted mutagenesis, cisgenesis and intragenesis. EFSA J..

[143] USDA Foreign Agricultural Service (USDA FAS) (2018). Russian Federation Agricultural Biotechnology Annual. GAIN Report Number: RS1833. https://apps.fas.usda.gov/newgainapi/api/report/downloadreportbyfilename?filename=Agricultural%20Biotechnology%20Annual_Moscow_Russian%20Federation_12-4-2018.pdf.

[144] OPOCE (2001). Directive 2001/18/EC of the European Parliament and of the Council of 12 March 2001 on the Deliberate Release into the Environment of Genetically Modified Organisms and Repealing Council Directive 90/220/EEC—Commission Declaration. Off. J. Eur. Union.

[145] OPOCE (2015). Directive (EU) 2015/412 of the European Parliament and of the Council of 11 March 2015 Amending Directive 2001/18/EC as Regards the Possibility for the Member States to Restrict or Prohibit the Cultivation of Genetically Modified Organisms (GMOs) in Their territory. Text with EEA relevance. Off. J. Eur. Union.

[146] OPOCE (2003). Regulation (EC) No 1829/2003 of the European Parliament and of the Council of 22 September 2003 on Genetically Modified Food and Feed (Text with EEA relevance). Off. J. Eur. Union.

[147] OPOCE (2003). Regulation (EC) No 1830/2003 of the European Parliament and of the Council of 22 September 2003 concerning the traceability and labelling of genetically modified organisms and the traceability of food and feed products produced from genetically modified organisms and amending Directive 2001/18/EC. Off. J. Eur. Union.

[148] CJEU, European Court of Justice Judgment in Case C-528/16. Organisms obtained by mutagenesis are GMOs and are, in principle, subject to the obligations laid down by the GMO Directive. PRESS RELEASE No 111/18. http://www.curia.europa.eu/.

[149] Bartsch D., Ehlers U., Hartung F., Kahrmann J., Leggewie G., Sprink T., Wilhelm R. (2020). Questions Regarding the Implementation of EU Mutagenesis Ruling in France. Front. Plant Sci..

[150] Council of the European Union (2019). Council Decision (EU) 2019/1904 of November 2019 Requesting the Commission to Submit a Study in Light of the Court of Justice’s Judgment in Case C-528/16 Regarding the Status of Novel Genomic Techniques Under Union Law, and a Proposal, if Appropriate in View of the Outcomes of the Study. Off. J. Eur. Union.

[151] European Commission EC Study on New Genomic Techniques. https://ec.europa.eu/food/plant/gmo/modern_biotech/newgenomic-techniques_en.

[152] European Commission Inception Impact Assessment: Legislation for plants produced by certain new genomic techniques. https://eur-lex.europa.eu/legal-content/EN/TXT/HTML/?uri=PI_COM:Ares(2021)5835503.

[153] Dong H., Huang Y., Wang K. (2021). The Development of Herbicide Resistance Crop Plants Using CRISPR/Cas9-Mediated Gene Editing. Genes.

[154] Zhang Y., Guo W., Chen L., Shen X., Yang H., Fang Y., Ouyang W., Mai S., Chen H., Chen S. (2022). CRISPR/Cas9-Mediated Targeted Mutagenesis of GmUGT Enhanced Soybean Resistance Against Leaf-Chewing Insects Through Flavonoids Biosynthesis. Front. Plant Sci..

[155] Karavolias N.G., Horner W., Abugu M.N., Evanega S.N. (2021). Application of Gene Editing for Climate Change in Agriculture. Front. Sustain. Food Syst..

[156] Smyth S.J. (2022). Contributions of Genome Editing Technologies Towards Improved Nutrition, Environmental Sustainability and Poverty Reduction. Front. Genome Ed..

[157] Gnanasekaran P., Ponnusamy K., Chakraborty S. (2019). A geminivirus betasatellite encoded βC1 protein interacts with PsbP and subverts PsbP-mediated antiviral defence in plants. Mol. Plant Pathol..

