# Supplementary material for: From Transgenesis to Genome Editing in Crop Improvement: Applications, Marketing, and Legal Issues
Source: Int J Mol Sci. 2023 Apr 12;24(8):7122. doi: 10.3390/ijms24087122 (PMC10138802; doi:10.3390/ijms24087122)
Supplement: Supplementary file 1 [file ijms-24-07122-s001.zip › ijms-2300612-supplementary.pdf]

Table S1. Crops improved through transgenic, cis/intragenic and GE approaches evaluated in field trials.

Trait	Crop	Gene	Source	Effect on gene expression	Effect/Phenotype	References
**Transgenic approach**						
**Herbicide-resistance**	Pineapple	*bar*	*S. hygroscopicus*	Expression	Enhanced tolerance to Glufosinate ammonium	[15]
**Abiotic stresses**	Wheat	*Hahb-4*	*H. annuus*	Overexpression	Enhanced drought tolerance	[19]
	Tomato	*cAPX*	Pea	Overexpression	Enhanced tolerance to heat, UV-B and sunscald stresses	[16]
	Chinese cabbage	*HTT2*	Arabidopsis	Overexpression	Enhanced resistance to heat	[17]
	Soybean	*GmMYB14*	Soybean cultivar Tianlon	Overexpression	Enhanced drought tolerance	[18]
**Biotic stress**	Rice	*cry1Ab*	*Bt*	Expression	Stem borer resistance	[26]
	Rice	*Xa21*	Wild *Solanum* species	Overexpression	Bacterial blight resistance	[25]
	Potato	*Rpi-vnt1.1*	*S. venturii*	Expression	Late blight resistance	[32]
	Potato	*Rpi genes*	Wild *Solanum* species	Expression	Late blight resistance	[33,34]
	Sugarcane	*Cry1Ab* and *EPSPS*	*Bt/-*	Expression	Cane borer resistance and glyphosate tolerance	[28]
	Sugarcane	*CP*	SCMV	Expression	SCMV strain E resistance	[29]
	Sugarcane	*CP*	SCYLV	Expression	SCYLV resistance	[30]
	Sugarcane	*CP*	SCMV	Expression	Lower SCMV disease incidence	[31]
	Papaya	*RP*	PRSV	Expression	High resistance or immunity against PRSV	[23]
	Sweet orange	*NPR1*	Arabidopsis	Overexpression	Enhanced Resistance against Huanglongbing	[24]

**Quality trait**	Wheat	*Wrinkled1a*<br>*DGAT1*<br>*Oleosin*	Maize<br>Arabidopsis<br>Sesame	Endosperm specific overexpression	Increased triacylglyceride levels	[38]
	Barley	*CKX*	Arabidopsis	Root-specific expression	Increased concentration of Zn in grains	[40]
	Soybean	*GmFAD2-1B*	Soybean	RNAi-mediated silencing	Improved oil quality	[35]
	Purple-fleshed sweet potato	*IbOr-Ins*	Sweet potato	Overexpression	Increased anthocyanins and carotenoids in storage roots	[36]
	Tomato	*PG*	Tomato	RNAi-mediated silencing	Improved shelf-life	[41]
	Canola	*DGAT1*	Arabidopsis and *B. napus*	Seed specific overexpression	Increased seed oil content	[39]
**Cis/intragenic approach**						
Cisgenesis						
**Biotic stress**	Potato	*Rpi*	Wild *Solanum* species	Expression	Late blight resistance	[52-54]
	Apple	*HcrVf1* and *HcrVf2*	Gene's own	Expression	Scab resistance	[57,58]
**Quality trait**	Barley	*HvPAPhy_a*	Gene's own	Overexpression	Improved grain phytase activity	[50]
Intragenesis						
**Biotic stress**	Apple	*HcrVf1* and *HcrVf2*	Own gene with different promoter and terminator	Overexpression	Scab resistance	[57]
**Quality trait**	Potato	*GBSS*	Gene's own	Silencing	High-amylopectin	[56]
	Potato	*StAst1*	Gene's own	Silencing	Reduced acrylamide level in French Fries	[55]
**Genome Editing approach**						
**Trait**	**Crop**	**Gene/s**	**Technology**	**Effect on gene expression**	**Effect/Phenotype**	**References**

**Yield/Yield related traits**	Rice	*Gn1a*, *DEP1, GS3* and *IPA1*	CRISPR/Cas9	Knock-out	Improved yield-related traits	[63]
	Rice	*BADH2, DEP1, Gn1a, QTL-GS3, QTL-GW2, Hd1, EP3, and LPA1*	CRISPR/Cas9	Knock-out	Improved yield-related traits	[64]
	Rice	*OsGS3*, *OsGW2* and *OsGn1a*	CRISPR/Cas9	Knock-out	Improved yield-related traits	[65]
	Rice	*SD1*	CRISPR/Cas9	Knock-out	Improved yield for plant height reduction	[66]
	Rice	*TaARE1*	CRISPR/Cas9	Knock-out	Improved yield for enhanced tolerance to N starvation and delayed senescence	[67]
	Maize	*ARGOS8*	CRISPR/Cas9	Knock-out	Improved drought tolerance and yield	[68]
**Abiotic stress**	Rice	*OsRR22*	CRISPR/Cas9	Knock-out	Improved salt stress tolerance	[69]
**Biotic stress**	Rice	*OsSWEET14*	CRISPR/Cas9	Knock-out	Enhanced bacterial blight resistance	[71]
		ERF922	CRISPR/Cas9	Knock-out	Enhanced rice blast resistance	[73]
		Bsr-d1, Pi21 and *ERF922*	CRISPR/Cas9	Knock-out	Enhanced rice blast and bacterial blight resistance	[72]
**Quality trait**	Wheat	*TaASN2*	CRISPR/Cas9	Knock-out	Decreased asparagine content	[80]
	Rice	*OsNramp5*	CRISPR/Cas9	Knock-out	Low Cd accumulation	[75,76]
	Rice	*OsNramp5 and OsLCT1*	CRISPR/Cas9	Knock-out	Low Cd accumulation	[77]
	Rice	*OsHAK1*	CRISPR/Cas9	Knock-out	Reduced Cs^+ uptake in conditions favoring high radiocesium uptake	[78]
	Maize	*waxy*	CRISPR/Cas9	Knock-out	Modified starch composition and improved yield	[74]
	Canola	*MYB28*	CRISPR/Cas9	Knock-out	Downregulation of *A-GSL* biosynthesis genes and reduction in accumulation of different glucosinolates	[79]
	Camelina	*FAD2*	CRISPR/Cas9	Knock-out	Increased seed oleic acid content	[81]

	Canola and Camelina	*BADC*	CRISPR/Cas9	Knock-out	Increased seed oil content	[82]

Genes: Ast1=asparagine synthetase (*Ast*)-*1*; ASNS=*asparagine synthetase; BADC= biotin/lipoyl attachment domain containing; BADH2=betaine aldehyde dehydrogenase; Bar=phosphinothricin acetyltransferase; cAPX=cytosolic ascorbate peroxidase; CP, coat protein; CKX=cytokinin oxidases/dehydrogenase*; *EP3=ERECT PANICLE; DEP1=dense and erect panicle 1; DGAT1= diacylglycerol acyl transferase 1; FAD2-1B=Delta-12 fatty acid desaturase 2 (FAD2); GBSS=granule bound starch synthase gene; Gn1a=grain number; GS3=QTL for grain length and weight; GW2=major QTL associated with rice grain width and weight;* HAK1=*Cs^+- permeable K^+ transporter*; *Hd1=heading date 1; HTT2=HEAT-INDUCED TAS1 TARGET2; LCT1=low affinity cation transporter 1*; *IbOr=Ipomoea batatas Orange gene; LPA1=loose plant architecture 1; NPR1=Pathogenesis Related genes 1; PG=Polygalacturonase;* PRSV= papaya ring spot virus; *RP=replicase; Rpi= Resistance genes against Phytophthora infestans; SD1=Semi-Dwarf1; are1=abnormal cytokinin response1 repressor1; Xa21= receptor-like kinase.*

Pathogens: Bt=Bacillus thuringensis; PRSV=papaya ring spot virus; SCMV= Sugarcane mosaic virus; SCYLV= Sugarcane yellow leaf virus.
